# In vitro comparative optical bench analysis of a spherical and aspheric optic design of the same IOL model

**DOI:** 10.1186/s12886-017-0407-5

**Published:** 2017-02-08

**Authors:** Tamer Tandogan, Gerd U. Auffarth, Chul Y. Choi, Stephanie Liebing, Christian Mayer, Ramin Khoramnia

**Affiliations:** 1 David J Apple International Laboratory for Ocular Pathology and International Vision Correction Research Centre (IVCRC), Department of Ophthalmology, University of Heidelberg, Im Neuenheimer Feld 400, Heidelberg, 69120 Germany; 2 Department of Ophthalmology, Kangbuk Samsung Hospital, Sungkyunkwan University School of Medicine, Kangbuk Samsung Hospital, Pyeong-dong, Jongno-gu, Seoul South Korea; 3 Eye Clinic, Klinikum rechts der Isar der Technischen Universität München, Munich, Germany

**Keywords:** Aspheric IOL, MTF, Optical quality, Strehl ratio, Spherical IOL

## Abstract

**Background:**

To analyse objective optical properties of the spherical and aspheric design of the same intraocular lens (IOL) model using optical bench analysis.

**Methods:**

This study entailed a comparative analysis of 10 spherical C-flex 570 C and 10 aspheric C-flex 970 C IOLs (Rayner Intraocular Lenses Ltd., Hove, UK) of 26 diopters [D] using an optical bench (OptiSpheric, Trioptics, Germany). In all lenses, we evaluated the modulation transfer function (MTF) at 50 lp/mm and 100 lp/mm and the Strehl Ratio using a 3-mm (photopic) and 4.5-mm (mesopic) aperture.

**Results:**

At 50 lp/mm, the MTF values were 0.713/0.805 (C-flex 570 C/C-flex 970 C) for a 3-mm aperture and 0.294/0.591 for a 4.5-mm aperture. At 100 lp/mm, the MTF values were 0.524/0.634 for a 3-mm aperture and 0.198/0.344 for a 4.5-mm aperture. The Strehl Ratio was 0.806/0.925 and 0.237/0.479 for a 3-mm and 4.5-mm aperture respectively. A Mann–Whitney *U* test revealed all intergroup differences to be statistically significant (*p* < 0.01).

**Conclusion:**

The aspheric IOL design achieved higher MTF values than the spherical design of the same IOL for both apertures. Moreover, the differences between the two designs of the IOL were more prominent for larger apertures. This suggests that the evaluated IOL provides enhanced optical quality to patients with larger pupils or working under mesopic conditions.

## Background

Irrespective of age, the regular healthy human cornea is attributable with a positive spherical aberration (SA). In young eyes, the negative SA of the crystalline lens usually counterbalances the cornea’s positive SA, resulting in low SA overall. But with aging, the lens’s SA changes from negative to positive, adding to the stable positive SA of the cornea and thereby decreasing the patient’s overall optical quality [1–3].

Conventionally, standard intraocular lenses (IOLs) used for cataract surgery or refractive lens exchange feature a spherical design. Similar to the aged natural lens, these spherical IOLs have a positive SA, which adds to the cornea’s positive SA. This results in reduced optical quality when compared to the juvenile natural lens. By comparison, implanting an aspheric IOL with a specific SA can lead to a significantly improved quality of vision for the patient, by modifying the overall ocular SA. These effects become more prominent with increasing pupil size, as SA is strongly dependent on aperture size. Numerous studies have proven the positive effect of aspheric IOLs on patients’ visual functions [4–7].

It is important to note, however, that other studies have demonstrated that there can be also very little to no significant difference between spherical and aspheric IOLs of similar or equivalent models [8, 9]. Several concepts of aspheric IOLs are available, such as IOLs with varying negative values of SA, “aberration neutral IOLs” (SA = 0 μm), and IOLs with a progressive change of SA from centre to periphery [10–13].

There are several external factors that can influence postoperative ocular SA, such as the change in corneal SA induced by surgical corneal incisions [14]. Therefore, it is of significant importance that aspheric IOLs are manufactured with little tolerance to deviations from the intended SA in order to avoid an additional source of error to the sum of residual SA.

Our study is intended to determine differences in some of the optical properties of the “aberration-neutral” aspheric IOL and the spherical IOL. To enable a meaningful comparison, we selected IOLs that shared the same manufacturer, material and dioptric power; the only variant was SA. We analysed the optical properties of the IOL models on the optical bench according to international standardised testing methods to provide objective data on established optical property parameters, such as the Modulation Transfer Function (MTF) and the Strehl Ratio [15]. All analyses were performed for two different aperture sizes (3 mm and 4.5 mm) to represent photopic and mesopic surrounding conditions.

In summary, this study is intended to provide a manufacturer-independent, objective evaluation of the “aberration-neutral” IOL’s potential to increase patients’ optical quality following lens replacement for cataract or refractive surgery.

## Methods

This prospective laboratory analysis was performed at the David J Apple International Laboratory for Ocular Pathology, University Eye Hospital Heidelberg, Heidelberg, Germany.

The analysis involved two groups of hydrophilic, acrylic, copolymer, one-piece, monofocal IOLs. Group 1 included 10 spherical IOLs (C-flex 570 C) and Group 2 included 10 aspherical IOLs (C-flex 970 C). Both lens types are manufactured by Rayner Intraocular Lenses Ltd., Hove, UK and are based on the same body design, featuring an optic diameter of 5.75 mm with a sharp edge design and an overall diameter of 12 mm. In this study, all analysed IOLs had a dioptric power of 26 D. The only difference between the IOLs was the SA value, resulting from slight differences in surface curvature.

The optical quality of all lenses was assessed using the OptiSpheric IOL PRO optical bench (Trioptics GmbH, Wedel, Germany) according to *ISO 11979–2, Ophthalmic implants - Intraocular lenses - Part 2: Optical properties and test methods.* This optical bench features different types of targets as objects, which are projected to the infinity through a collimator. As a result, the tested lens provides an image of the target at its focal plane. Composed of a microscope objective and an imaging system conjugated with a CCD camera, the measurement head scans through the imaging zone to find the best focus image created by the tested IOL. The image detected by the camera is then used for further analysis. The light source illuminating the target is a broad band visible spectrum light source associated with a narrow band interference filter at 546 nm, in accordance with the ISO norm. Narrow band light is appropriate when comparing the two IOL models because they are made of the same material having the same chromatic aberration. The image obtained via the IOL is collected by a microscope and analysed by the integrated software. This method resonates with previous bench tests that have been used to analyse optical properties of different monofocal or multifocal aspheric IOLs [15–17].

In our setting, a thin line of light was chosen as a test target in order to obtain the Line Spread Function (LSF), from which the MTF and Strehl Ratio were calculated. The MTF of an optical system describes the amount of contrast that is passed through the system for a given spatial frequency or object size; it is defined as the amplitude of the image contrast divided by the amplitude of the object contrast and is a function of spatial frequency. The contrast decreases more rapidly at higher spatial frequencies (i.e. the number of line pairs [lp] per millimetre) or with object size. The MTF therefore represents the capability of an optical system to transfer the details of an object into an image. The Strehl Ratio enables the comparison of the maximum aberrated image intensity from a point source to the maximum achievable intensity using an ideal, diffraction-limited optical system. As such, the Strehl ratio is closely associated with quality of vision [18].

For testing, each IOL was placed within a model eye system. The haptics of the samples were rotationally oriented at random to compensate for the single slit measurement. The model cornea was an aberration-free achromatic doublet – as specified in ISO 11979–2 – so that any aberration observed or the effect of any aberration on image quality would result only from the IOL. In each instance, the IOL was placed in an IOL holder (11.0–13.0 mm) before being inserted into the model eye, which was filled with deionized water. Each IOL was positioned so that the lens’s anterior side faced the incident light, and the IOL holder guaranteed tilt-free orientation of the lens during the testing procedure. The collimated light passing through the artificial cornea was focused on the IOL, thereby simulating the vergence of a human eye, and the device could automatically detect the optical axis of each IOL. Measurements were performed at ambient temperature as recommended by the ISO standard because the IOL dimensions do not deviate appreciably from those under in situ conditions.

The through-focus MTFs were measured using 3.0 mm and 4.5 mm apertures at 50 lp/mm and 100 lp/mm in the model eye. The focus was shifted gradually from an object at infinity to increasingly closer distances. The two apertures of 3.0 mm and 4.5 mm were chosen to correspond with photopic and mesopic surrounding conditions, while the spatial frequency of 50 lp/mm corresponded with the fundamental frequency of the 20/40 line on the Snellen eye chart. The Strehl Ratio was measured using the same 3.0 mm and 4.5 mm apertures.

Each lens underwent three measurement cycles without positional alteration to obtain all outcome metrics. This resulted in a mean value for each metric of each lens. Medians of the resulting ten mean values per group were then used for further statistical analysis. A Mann-Whitney *U* test was performed to detect differences in optical property metrics between groups.

## Results

**Fig. 1:**
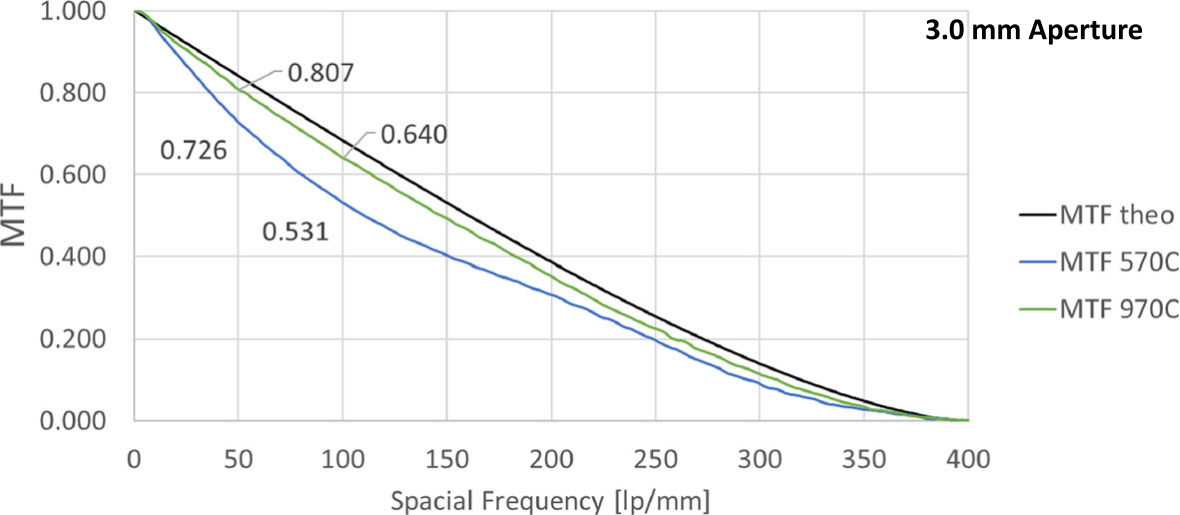
Modulation Transfer Function: One example of each intraocular lens at 3.0 mm aperture. *Black line*: Theoretical upper limit, *blue and green*: Measured MTFs for both evaluated lenses

**Fig. 2:**
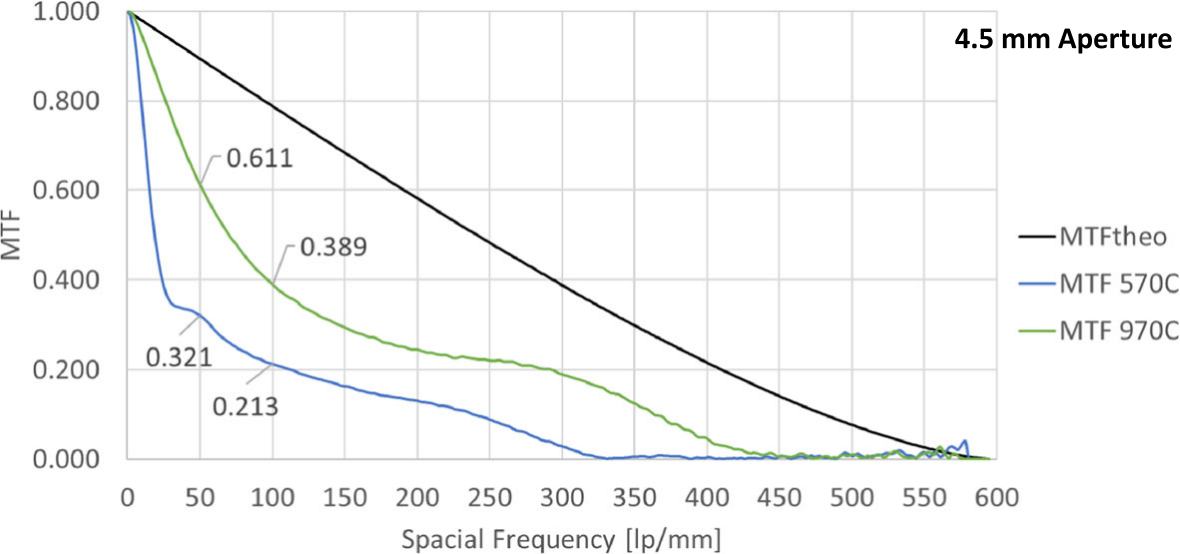
Modulation Transfer Function: One example of each intraocular lens at 4.5 mm aperture. *Black line*: Theoretical upper limit, *blue and green*: Measured MTFs for both evaluated lenses

Figures 1 and 2 show typical examples of the MTF values for 3.00 mm and 4.5 mm apertures at all spatial frequencies for one IOL per model. Tables 1 and 2 summarise the MTF results for 3 mm and 4.5 mm apertures at 50 lp/mm and 100 lp/mm. The results demonstrate that for all measurements comparing the spherical and aspheric models, values for the aspheric IOL were always significantly higher (*p* < 0.01).

**Table 1:** Modulation Transfer Function values at 3-mm aperture

MTF	Spherical IOL	Aspheric IOL	*P*-Value
50 lp/mm Median (Range)	0.7133 (0.6900 to 0.7310)	0.8058 (0.7970 to 0.8130)	0.0002*
100 lp/mm Median (Range)	0.5265 (0.4980 to 0.5430)	0.6345 (0.6230 to 0.6450)	0.0002*

*Statistically significant difference (*p* < 0.05, Mann-Whitney-*U* test)

**Table 2:** Modulation Transfer Function (MTF) values at 4.5-mm aperture

MTF values	Spherical IOL	Aspheric IOL	*P*-Value
Median (Range) at 50 lp/mm	0.2970 (0.2790 to 0.3280)	0.5935 (0.5370 to 0.6880)	<0.0001*
Median (Range) at 100 lp/mm	0.1975 (0.1880 to 0.2050)	0.3445 (0.3230 to 0.3920)	<0.0001*

*Statistically significant difference (*p* < 0.05, Mann-Whitney-*U* test)

Tables 3 and 4 summarise the Strehl Ratio values for 3 mm and 4.5 mm apertures, which also show that values for the aspheric IOL were significantly higher in all instances (*p* < 0.01).

**Table 3:** Strehl Ratio values for 3 mm aperture

Strehl Ratio	Spherical IOL	Aspheric IOL	*P*-Value
Median	0.8143	0.9288	0.0002*
Minimum	0.7630	0.8800	
Maximum	0.8450	0.9410	

*Statistically significant difference (*p* < 0.05, Mann-Whitney-*U* test)

**Table 4:** Strehl Ratio values for 4.5 mm aperture

Strehl Ratio	Spherical IOL	Aspheric IOL	*P*-Value
Median	0.2355	0.4870	0.0002*
Minimum	0.2260	0.4430	
Maximum	0.2910	0.5080	

*Statistically significant difference (*p* < 0.05, Mann-Whitney-*U* test)

## Discussion

Ocular SA has a significant influence on patients’ quality of vision. Implanting specific aspheric lenses according to the intended optical use can help to optimise quality of vision and certain visual functions. Currently, there are two main approaches that are adhered to in practices. In the first approach (which has become widely accepted), an IOL with negative SA is selected in order to reduce overall ocular SA. Numerous studies prove the benefit of such IOLs for patients with large pupils, mainly at mesopic conditions [4, 5, 7]. The second approach entails the implantation of “aberration-neutral” IOLs, which are intended to avoid adding further SA to the existing ocular SA – namely that of the cornea [6].

In this study we have intended to provide an objective analysis of the potential optical benefit achievable with an “aberration-neutral” IOL. To exclude as many co-variants as possible, we chose a study design which specifically examined IOLs developed by the same manufacturer, of the same material and of the same design, differing only in SA. Moreover, the study entailed a highly standardised analysis of optical properties, following the internationally-recognised ISO 11979–2 norm for testing optical systems.

The adopted model eye approach enabled a high level of control of the surrounding conditions: from corneal spherical aberrations to aperture sizes corresponding with pupil sizes at photopic and mesopic conditions to imaged spatial resolution, simulating tasks requiring different visual acuities. Consequently, this approach allowed us to show the tested IOLs’ inherent ability to improve optical quality without other factors interfering with results (such as subjective perception). However, the aberration free model cornea of ISO 11979–2 is different from the mean human corneal SA, which is a limitation of this laboratory study.

The two main objective outcome metrics, the MTF and Strehl Ratio values, were chosen in accordance with the ISO 11979.2 norm. Optical bench evaluations, such as MTF testing, provide valuable information on the optical quality of IOLs. To accommodate varying surrounding conditions and to evaluate the possible optical benefits under these different conditions, we tested each IOL at 50 lp/mm and 100 lp/mm spatial resolutions (representing visual acuities of 20/40 and 20/20 respectively) and two aperture sizes (3.0 mm and 4.5 mm, representing photopic and mesopic surrounding conditions). The test conditions therefore represent clinically relevant visual tasks: seeing small details and seeing in mesopic conditions.

Overall, the MTF values were higher at all spatial resolutions for the aspheric IOL compared to the spherical IOL (Figs. 1 and 2). The aspheric IOL design achieved significantly higher MTF and Strehl Ratio values than the spherical design at the two selected and visually most relevant spatial resolutions and for both apertures. The differences between the aspheric and spherical design were more prominent in mesopic conditions. This suggests that patients with larger pupil sizes and/or working in mesopic conditions might benefit more from an aspheric IOL design than a spherical one. The larger difference in MTF at 4.5 mm pupil is expected from optical theory, since SA is highly aperture dependent. Our findings correspond with subjective evaluations of quality of vision after aspheric IOL implantation performed in previous studies [5, 6]. However, these benefits are reported to become relevant in terms of quality of vision for pupil sizes of about 5 mm or larger only [5]. Our study was able to show significantly improved optical properties with the aspheric IOL when increasing the pupil size from 3.0 mm to 4.5 mm in comparison to the spherical IOL. The aspheric IOL also performed better than the spherical one under photopic conditions. These findings suggest that even patients with smaller pupil sizes, such as elderly cataract patients, might benefit from “aberration-neutral” aspheric IOLs, provided the lens has been chosen considering the existing corneal SA. However, individualising aspheric IOL selection to corneal SA is challenging, as several other factors are known to alter postoperative ocular SA. Amongst others, these contributing factors include surgically induced SA as a result of a change in corneal curvature due to the cataract incision as well as the fluctuations of SA measurements within same individual [14]. Various clinical studies have demonstrated little to no statistical significance between aspheric and spherical models of an IOL [8, 9].

## Conclusions

In summary, it can be concluded that patients with larger pupil sizes and/or working in mesopic conditions might benefit more from an aspheric IOL design. Additionally, patients with smaller pupils or individuals working in photopic conditions may yield better results in terms of quality of vision with an aspheric IOL design.

## Abbreviations

IOL: Intraocular lens
IOLs: Intraocular lenses
LSF: Line Spread Function
MTF: Modulation Transfer Function
SA: Spherical aberration
